# A Novel Microfiber Wipe for Delivery of Active Substances to Human Skin: Clinical Proof of Concept

**DOI:** 10.3390/polym12112715

**Published:** 2020-11-17

**Authors:** Martin Kaegi, Christian Adlhart, Markus Lehmann, Marius Risch, Werner Wessling, Peter Klaffenbach

**Affiliations:** 1 Haut Zentrum Zürich AG, Schaffhauserstrasse 355, 8050 Zürich, Switzerland; praxis.kaegi@hin.ch; 2 Institute of Chemistry and Biotechnology, Zurich University of Applied Sciences ZHAW, Einsiedlerstrasse 31, 8820 Wädenswil, Switzerland; adas@zhaw.ch; 3 FILAG Medical Schweiz AG, Schweizersbildstrasse 41, 8207 Schaffhausen, Switzerland; m.risch@filag-medical.ch (M.R.); werner@wessling-online.com (W.W.); pklaffenbach@web.de (P.K.)

**Keywords:** microfiber, skin cleansing, topical delivery, Raman spectroscopy, liposomes, niacinamide

## Abstract

A novel technology for the delivery of active substances to the skin based on microfibers loaded with dried active substances was developed. The objective of this work was to demonstrate deposition of the active substances on the skin including concurrent cleansing properties of the wipe. As model active substance to measure deposition capacity Niacinamide was used and as parameter to measure cleansing capacities of the wipe squalene uptake was measured. Wipes loaded with niacinamide were used in the face and the forearm of 25 subjects. By means of Raman spectrometry the deposited niacinamide was analyzed before and after application. Wipes used on the face were analyzed for squalene to assess skin cleansing properties and for residual niacinamide. Forearm analysis including placebo and verum on left and right arm respectively was performed to rule out changes of the skin through application of the tissue. Measured amounts of niacinamide from face application demonstrate statistically significant results in the study population. Analysis of the wipes used show a liberation of 28.3% of niacinamide from the wipes and an uptake of 1.7 mg squalene per wipe. Results from forearm application show statistically significant differences (*p* < 0.05) between placebo and active for the complete study population. Sub group analyses are significant for both gender and ethnicity for face and forearm analysis respectively. Results clearly demonstrate deposition of niacinamide on the skin and the cleansing properties of the wipe. The institutional review board approved this prospective study.

## 1. Introduction

In general, the topical delivery of active substances can be differentiated in terms of carrier technology (delivery technology) for active ingredients and the delivery of active ingredients as such [1,2].

In topical delivery the carrier technologies are almost exclusively based on aqueous systems, which themselves require almost always several additives for stability of the aqueous solutions and to avoid microbial contamination [3]. Most of the chemical additives in these carrier systems are not chemically inert, resulting in complex galenic evaluations requiring time and money for the development [4].

These additives, such as preservatives, alcohol, emulsifiers, fragrances, stabilizers and silica increase the risk of adverse reactions of the skin [5].

Cleansing of the skin, normally done prior to the treatment of the skin, is typically based on chemical cleansers, such as soaps and alcohols or oil based liquids. All of them are increasing the risk of adverse reactions of the skin even further [6].

Use of liposomes has been introduced in skin care products. This relatively novel approach for the delivery of active compounds to the skin involves tiny vesicles formed by monolayer or bilayer membranes manufactured using phospholipids and high pressure homogenization. The main compound of lecithin, phosphatidylcholin, helps the skin to reinforce its barrier function and enhances skin moisture [7]. Additionally, using liposomes some restrictions like limited penetration, poor solubility or stability and other characteristics can be improved [8].

The present study is run on a novel dermal delivery system for skin care that combines wipes built of a three-dimensional microfiber structure with dried, liposomised and dermatologically useful active ingredients (i.e., niacinamide) [9,10,11]. As it is dry, it does not require any additives. For application, the wipe is moistened with water and then used to massage the skin for about 45 s. During use, the three dimensional structure of the wipes provide mechanical cleansing, including soft peeling, while at the same time releasing the actives it holds to the skin. No further products are required before or after application of the wipe. There are patents granted or pending in all relevant global markets including US [11], Europe [9,10], India [12], China [13] and Japan [14].

The technology as such replaces thereby cleansers and creams or any other conventional delivery technology and opens abundant possibilities for future cosmetics, medical devices and drug development with advanced and convenient delivery systems.

The wipe is a mechanically refined, knit-warped, three dimensional textile with strong drug uptake capacities based on Polyester (PES) and Polyamide (PA). Two multi-filament yarns are used to warp knit a fabric precursor of less than 180 g/m^2^ with a PES/PA ratio of 60/40, 80% of the fibers being microfibers and having a diameter of less 50 µm and weighing between 150–200 dtex. One side is then mechanically roughened to create a fluffy surface on the respective side. The fluff is cut resulting in a soft, yet short haired and compact surface. The fabric is then being subject to an alkaline hydrolysis, combined with a thermal mechanical treatment to chemically split the microfibers and dissolve a part of the polyester yielding in a strong increase of absorption capacity of 4–7 times its weight, with a predefined surface weight and pH-value of approximately 5.5 and a defined PES/PA ratio of approx. 60/40. The fabric is then being loaded with liposomized active substances by immersion in a liquid solution then being dried. The resulting fabric is completely dry as water and alcohol completely evaporate during the drying process. The drying process does neither decompose liposomes nor niacinamide [15]. As for liposomes, once dry the liposomal membrane will disperse but will reconstitute when moistened prior to use of the wipe. The active ingredient i.e., niacinamide is distributed in the inner and in the outer phase of a liposome, both before drying and after reconstitution [16]. Drying of liposomes is quite common in the pharmaceutical industry to enhance stability.

**Figure 1:**
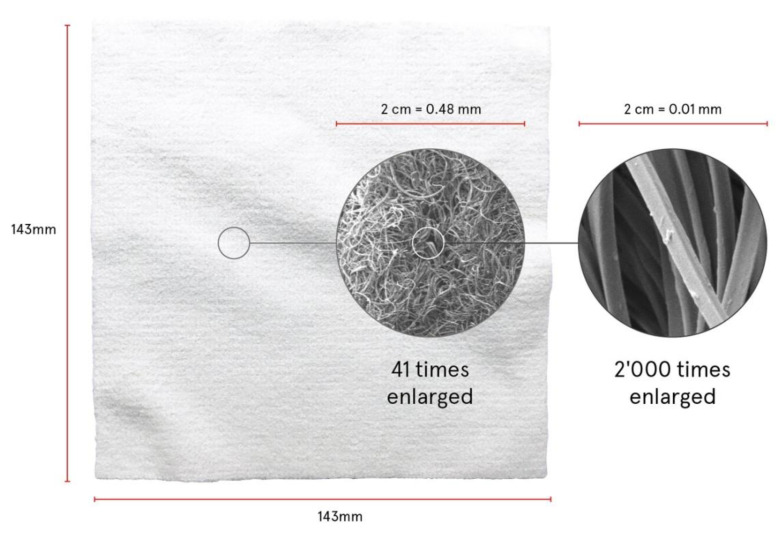
Microfiber wipe in total and magnifications of filaments.

When moistened before use, the active substances disperse in water but remain in the wipe through the capillary capacity of the textile structure. The application on the skin presses water and dispersed substances onto the skin thus liberating active substances from the wipe. While water vaporizes lecithin and the active substances remain on the skin. At the same time, the microfiber structure of the wipe cleanses and softly exfoliates the skin. Figure 1 shows the wipe and the structure of the filaments in different magnifications. 

Under the brand name “Filabé of Switzerland” the technology is already successfully introduced into the market with cosmetic ingredients. 

The present study was purely designed to demonstrate the deposition of the active substance and the cleansing effect of the microfiber wipe. Further the study should prove that the deposited ingredients are not reabsorbed by the wipe during cleansing. 

To demonstrate the delivery and the remaining a two-fold approach was selected: By means of Raman spectrometry the presence of niacinamide on the skin shall be detected. Niacinamide was selected because of its visibility by Raman spectroscopy and because of the fact that it is widely and increasingly used in cosmetic and also medical products e.g., to decrease transepidermal water loss (TEWL) [17].

Raman spectrometry is a well established method for the in-vivo determination of topically applied substances [18,19,20]. Therefore this method was selected for the demonstration of the depositing capacity of this unique technology. In addition to the demonstration of the depositing of niacinamide on the skin the amount of niacinamide delivered by the wipe shall be assessed by comparing the residual content of niacinamide in the used wipe with the typical content of unused wipes. A method for the quantitative analysis of niacinamide in the wipe by means of high performance liquid chromatography combined with ultraviolet detection (HPLC-UV) has been developed and validated for use as quality control instrument.

Facial skin was selected for the determination of niacinamide deposition. Raman measurements are conducted before and after use of the wipe. To exclude any potential effects of the microfibers on the skin (e.g., abrasion) influencing Raman signals, a comparison of Raman measurements on the volar forearms using placebo and active wipes is performed as validation.

Cleansing of the skin in the cosmetic literature is defined as removing dirt, sebum, sweat, exfoliated corneocytes, exudates and other non-wanted substances from the skin [21]. Based on this definition a number of ways to demonstrate the cleansing effect of the microfiber wipe are possible, including but not limited to detection of human skin cells by means of DNA testing, detection of sebum substances, detection of skin lipids, detection of skin-typical microorganisms. Confirmation of human cells as well as skin-typical microorganisms is rejected because only a very simple present or non-present conclusion could be obtained. Analysis of skin lipids in the used wipe is deemed difficult due to the presence of significant amounts of phospholipids (lecithin) from the liposomes. Human sebum is composed of approx. 30%–50% lipids, free fatty acids 15%–30%, wax esters 26%–30%, squalene 12%–20%, cholesterol esters 3%–6%, and cholesterol 1.5%–2.5% [22]. Analysis of squalene from fatty matrices is well established by means of gas liquid chromatography usually combined with mass spectrometry (GLC-MS) [23]. Development of a combined method for the analysis of squalene and niacinamide from the wipe was deemed feasible. Therefore, squalene is selected as the parameter to demonstrate the cleansing effect of the wipe by means of confirmation of human skin compounds in the used wipe.

## 2. Materials and Methods 

### 2.1. Study Population

Subjects were enrolled from the general population of Schenefeld/Hamburg (Germany) and the neighboring communities. All subjects had a complete understanding of the test procedure. An informed consent was prepared explaining the product under investigation and the investigation to be performed in full detail. The subjects were entitled to withdraw from the study at any time. All subjects gave their informed consent for inclusion before they participated in the study. All subjects gave prior informed consent for inclusion in the study. The study was conducted in accordance with the Declaration of Helsinki, and the protocol was approved by the Institutional Review Board of proDERM GmbH (Schenefeld, Germany) with project identification code 2020/0017 on October 21st, 2020. There was no drop out; all 25 subjects completed the study. No adverse events have been observed. Inclusion criteria included written informed consent to participate in the study, willingness to actively participate in the study and to come to the scheduled visits, male or female, 20 to 60 years of age, BMI < 30, uniform skin color and no erythema or sun tanning in the test area. The following exclusion criteria were applied: Female subjects: Pregnancy or lactation, drug addicts, alcoholics, AIDS, HIV-positive or infectious hepatitis, conditions which exclude a participation or might influence the test reaction/evaluation, participation or being in the waiting period after participation in cosmetic and/or pharmaceutical studies pertaining to the test area, active skin disease at the test area, signs of acne and/or skin irritation, documented allergies to cosmetic products and/or ingredients, insulin-dependent diabetes mellitus, cancer not being diagnosed as cured and requiring chemotherapy, irradiation and/or hormonal treatment within the last 2 years, one of the following illnesses with reduced physical capability/fitness: asthma (symptom-free allergic asthma is not an exclusion criterion), hypertension, cardiovascular diseases, wounds, moles, tattoos, scars, irritated skin, excessive hair growth, etc. at the test area that could influence the investigation, freshly sun-tanned skin in the test areas, regular use of tanning beds, any topical medication at the test area within the last 7 days prior to the start of the study, therapy with retinoid containing drugs (topical or non-topical) within the last 4 weeks prior to the start of the study, systemic therapy with immunosuppressive drugs (e.g., corticosteroids) and/or antihistamines (e.g., antiallergics) within the last 7 days prior to the start of the study.

13 Caucasian subjects, 6 Asian subjects (from east or west Asia and at least one parent with Asian ethnicity), 6 dark skin subjects (Fitzpatrick V/VI) were enrolled in the study. Overall study population age was 21 to 59, median 49, 13 male subjects aged 21 to 58 (median 49) and 12 female subjects aged 22 to 59 (median 49.5).

In order to exclude any influence of the wipe on the skin that could affect Raman spectroscopy (e.g., abrasive effects of the wipe), a comparison between placebo and active was performed in a double blind set up. One forearm was treated with a placebo wipe, the other forearm with niacinamide loaded wipe. After treatment analysis by Raman spectrometry was performed. Raman spectra were analyzed using the proprietary software supplied by the instrument manufacturer.

All subjects were instructed to obey the following rules prior to start of the study: 4 h prior to the study no washing or rinsing washing in the application area was allowed. No cosmetic or cleansing products were allowed in the application area for 24 h prior to the study and no topical drugs for 7 days. Male volunteers may have been shaved electrically or wet but not less than 24 h prior to the study and last shaving not further than 3 days prior to the study. No use of aftershave or perfume 24 h prior to the study. 

Facial application test procedure: 15 mL tap water was added to one wipe, each wipe is folded twice to obtain 4 squares before application of water. First quarter to be used on forehead (including temples), second quarter on right side of face down to chin, third quarter on left side of face down to chin, and fourth quarter nose and eyelids and chin. The wipe was swiped with a frequency of approx. 2 swipes per second with the following number of replicates: 7 on forehead and each cheek, 6 on chin, upper lip and nose and 4 on the eyelids. To ensure maximal reproducibility the complete application on all volunteers was performed by an experienced and specifically trained operator. This protocol was developed based on the average of relevant user experience of Filabé products, including a survey of 31, thereof 20 female people having used Filabé for more than 2 months. The questions asked were the total number of movements on each specific area of the face, the pressure applied (soft, medium, heavy) as well as the process flow. 

After application all wipes from facial application were collected in closable bottles for later analysis. The used wipes were stored and shipped to the laboratory for chemical analysis at 5 °C.

Forearms application test procedure: Each wipe was moistened using 15 mL of tap water after folding it in quarters. 8 swipes with a frequency of approx. 2 swipes per second covering an area of 7 × 10 cm marked prior to application. For best reproducibility, all applications were performed by the same experienced operator. 

### 2.2. In-Vivo Test by Means of Confocal Raman Spectroscopy

Raman spectra were obtained using a confocal Raman spectroscopy device (gen2-SCA Ultimate, RiverD International B.V., Rotterdam, The Netherlands). The Raman spectroscopy device was equipped with an oil-immersion object lens and a 785 nm excitation laser for acquiring fingerprint profiles with wavenumbers from 400 to 1800 cm^−1^.

Raman spectra were obtained using the 50 µm pinhole with an axial resolution of 5 µm and an integration time of 5 s. Measurements were performed at the skin’s surface (0 µm) and at 5 µm in the skin. The measurements were repeated approximately 8 times in the test area of 500 × 500 µm. The obtained spectra were analyzed by Skintools 3.2 software (RiverD International B.V., Rotterdam, The Netherlands). The algorithm calculated the content of topically applied components in the stratum corneum. The method used spectral fitting of in vivo skin spectra with the default fit model, consisting of Raman spectra of intrinsic stratum corneum components, and one or more added spectra of topically applied components, in case of the present study spectra of liposomized niacinamide. No calibration using quantified reference spectra was performed. The results are presented in arbitrary units (i.e., niacinamide [a.u]).

**Figure 2:**
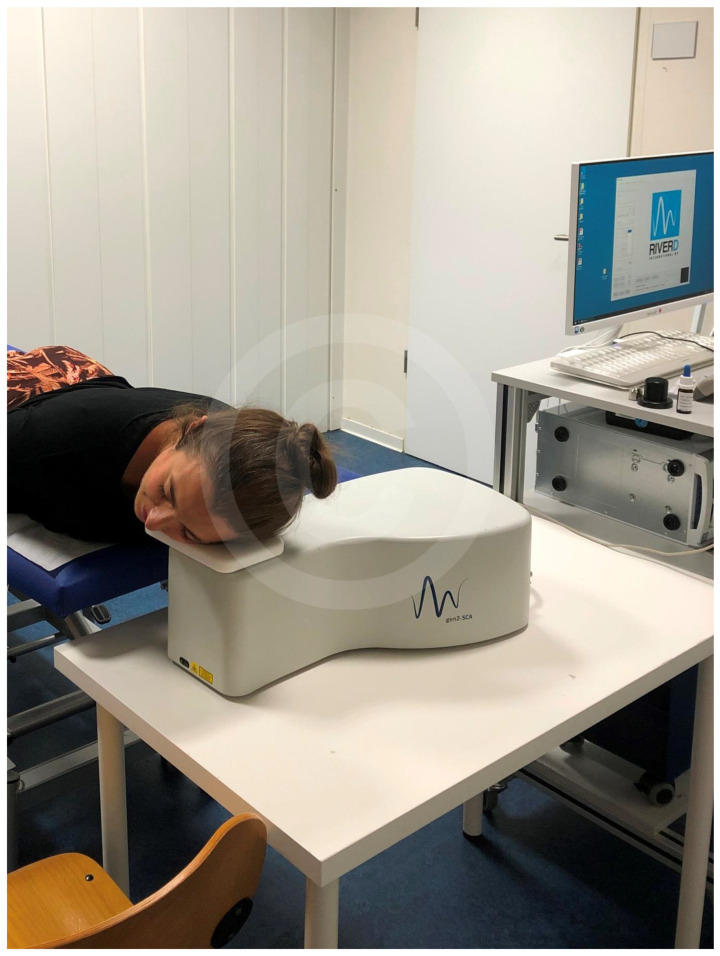
Measurement by Raman-Spectrometry (Copyright proDERM).

Figure 2 shows the testing on the face of a volunteer. 

Raman measurements were performed in an air-conditioned room at a temperature of 22 ± 2 °C and at 50 ± 7.5% relative humidity. The subjects were in the climatized room for at least 30 min prior to commencing the study.

### 2.3. Microfiber Wipes 

Microfiber wipes were obtained from regular production of Filabé wipes (Filabé of Switzerland Ltd., Schaffhausen, Switzerland) prior to loading with liposomized active substances. The microfiber wipe was loaded with liposomized niacinamide 6% obtained from Mibelle AG (Buchs, Switzerland). The solution was composed of 2% lecithine, 15% ethanol (96%), 6% niacinamide, and 77% water. Ethanol and water were removed during the drying process. The loading process was performed at Cilander AG (Herisau, Switzerland) using a lab scale coating machine Type HF41188 obtained from Werner Mathis AG (Oberhasli, Switzerland). The pressure of the squeezing roller was set to obtain a pickup (weight gain of the coated wipes) of at least 70%. After loading the wipes were dried for 2 min at 110 °C in a lab dryer. Placebo wipes were obtained directly from production material from the same batch as active without loading with liposomized niacinamide. There was no visible difference between active and placebo wipes.

### 2.4. Post Use Chemical Analysis for Squalene and Nicinamide 

The used wipes from facial use were analyzed for niacinamide and squalene by ILB (Interlabor Belp, Belp, Switzerland). Each wipe was cut into 4 approximately equal parts and weighted into a 100 mL screw cap bottle obtained from Schott (Mainz, Germany). 50 mL 0.01 M phosphoric acid obtained from Merck (Zug, Switzerland) was added. The bottle was closed and shaken for approx. 10 min on a horizontal shaker. 50 mL of extraction solvent was added. The extraction solvent was prepared by weighing of 25 mg squalane (internal standard) obtained from Sigma-Aldrich (Buchs, Switzerland), in a 50 mL volumetric flask, and dissolved in pentane, HPLC grade obtained from Sigma-Aldrich (Buchs, Switzerland). The volumetric flask was made up to volume using the same solvent. 5 mL of this solution was diluted with pentane to 250 mL to obtain the extraction solvent (concentration of squalane 10 µg/mL). After addition of the extraction solvent the bottle was shaken again for approx. 10 min on a horizontal shaker. For analysis of squalene an aliquot of the organic phase (upper layer) was removed and put into a GC vial. For analysis of niacinamide 2.5 mL of the aqueous phase (lower layer) was diluted to 20 mL with 0.01 M phosphoric acid obtained from Merck (Zug, Switzerland). The solution was then is filtered through a 0.45 µm nylon filter into a HPLC vial.

Analysis of niacinamide was performed by means of reversed phase high performance liquid chromatography combined with ultraviolet detection (HPLC-UV). Mobile phase A was prepared by mixing of 10 mL diluted acetic acid R obtained from Honeywell (Aesch, Switzerland) (As defined in the reagents section of the European Pharmacopeia) and 1800 mL water. 60 mL of diluted ammonium hydroxide R3 obtained from Honeywell (Aesch, Switzerland) (As defined in the reagents section of the European Pharmacopeia) and 30 mL acetonitrile obtained from Honeywell (Aesch, Switzerland) was added. Water was added to obtain 2000 mL. Mobile phase B was prepared by mixing 500 mL mobile phase A with 500 mL acetonitrile obtained from Honeywell (Aesch, Switzerland). A HPLC column X-Bridge 150 × 4.6 mm, 3.5 µm particle size was used, obtained from Waters (Baden-Dättwil, Switzerland). Flow rate was set to 1 mL/min. The UV detector was operated at 264 nm. The column temperature was maintained at 25 °C. 5 µL of the sample was injected. Solvent composition was 98% A/2% B from 0 to 2 min, followed by a linear gradient to 0% A/100% B for 14 min. 98% A/2% B was restored at 17 min and maintained until minute 25 to re-equilibrate the system prior to next injection. 

An Agilent 1100 HPLC System obtained from Agilent (Basel, Switzerland) was used. Quantification was performed using the external standard method based on niacinamide reference standard obtained from Sigma-Aldrich (Buchs, Switzerland). 

**Figure 3:**
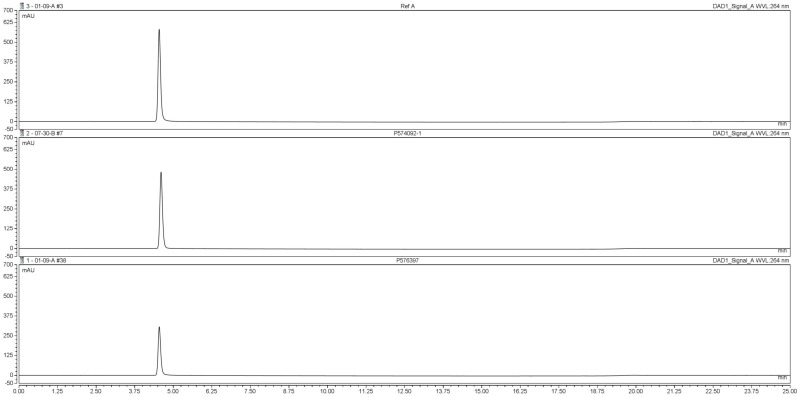
HPLC chromatograms of niacinamide reference solution (**top**), sample solution from quality control (**middle**) and sample solution from used wipe (**bottom**).

Figure 3 shows chromatograms of the reference solution containing 500 µg/mL niacinamide and typical sample solutions. Retention time of niacinamide was approx 4.5 min. The sample chromatogram was free of interfering peaks.

The method was validated for selectivity, linearity (0.005–0.505 mg/mL, r^2^ = 0.9999), and recovery.

Analysis of squalene was performed by means of capillary gas liquid chromatography coupled with electron impact mass spectrometry (GC-MS). An Agilent 78090B/MSD5977B GC-MS System obtained from Agilent (Basel, Switzerland) was used. For separation a DB-35MS, 30 m × 0.25 mm, 0.25 µm film capillary column obtained from Agilent (Basel, Switzerland) was used. Helium was used as carrier gas with a constant flow of 1.5 mL/min. 2 µL of sample was injected by means of an automatic sampler using pulsed splitless injection at 250 °C (250 kPa for 0.5 min). Separation was performed using the following temperature program: 160 °C maintained for 0.5 min, starting a temperature ramp at 10 °C/min to 320 °C. Detection was performed after electron impact ionization (EI) with 70 eV using selected ion monitoring (SIM) of m/z 57, 69, 85, 95. Ions at m/z 69 and 95 were selected to detect squalene [24]. Ions at m/z 57 and 85 were selected to detect the internal standard squalane [25]. Quantification was performed using the internal standard method using squalane as internal standard. Squalene reference standard obtained from Sigma-Aldrich (Buchs, Switzerland) was used. 

**Figure 4:**
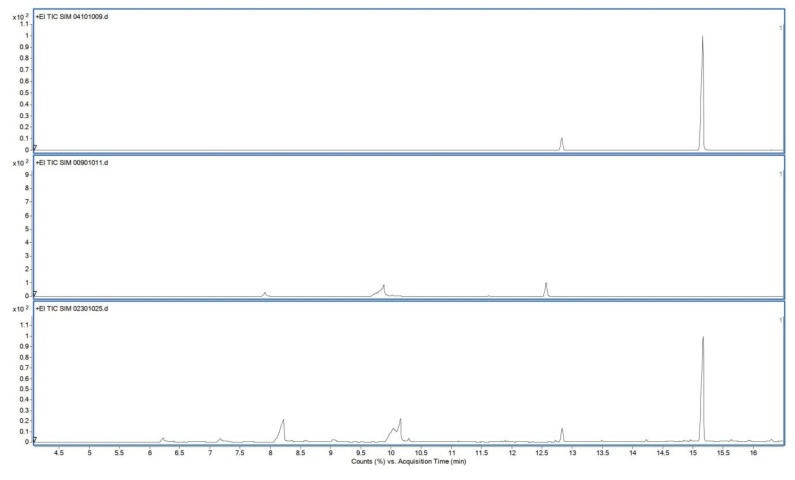
GC-SIM total ion chromatograms of reference solution (**top**), sample solution from unused wipe (**middle**) and sample solution from used wipe (**bottom**).

Figure 4 shows typical chromatograms of the reference solution containing 90 µg/mL squalene (top), typical sample solution from unused wipe (middle) and typical sample solution from used wipe (bottom). Retention times of squalene and squalane (internal standard) were approx 15 and 12.8 min, respectively. Chromatograms of pentane and unused wipes were free of interfering peaks at the retention time of squalene. The internal standard squalane was well separated from the analyte squalene.

The method was validated for selectivity and linearity (1–90 µg/mL r^2^ = 0.992).

Statistical data evaluation was performed using PSPP version 1.2 by Ben Pfaff (www.gnu.org/software/pspp).

## 3. Results

### 3.1. Deposition of Niacinamide on Facial Skin

As described in the materials and methods section at each test point two measurements are performed: At the skin’s surface (0 µm) and at 5 µm in the skin respectively. The results calculated by the skintools software at each measurement point were summarized and the mean of all measurement points was calculated and statistically evaluated.

Prior to application a mean result of 0.68 is calculated (N = 25, SD = 3.39), values range from 0 to 16.97. It is noteworthy that only one subject showed a result different from 0 (subject #22 male, 32 years, African). After application of the loaded wipe a mean result of 49.77 was calculated (N = 25, SD = 29.64), values ranged from 0 to 117.50. Only subject #9 (female, 22 years, African) showed a result of 0 after application.

### 3.2. Analysis of Residual Niacinamide Content in Wipes Used on the Face

The 25 wipes used in the face of the volunteers were analyzed for residual niacinamide content. Prior to conducting the study representative samples of the wipes were analyzed using the same method to determine the initial content of niacinamide. Results of the initial content were 169.5 mg/wipe (n = 3, RSD 1.64%). After use a mean of 121.5 mg niacinamide/wipe was determined (n = 25, RSD 5.07%), values ranged from 106.6 6 to 130.4 mg/wipe. Compared to the initial content a mean of 28.3% was liberated from the wipes (RSD 12.8%), minimum 23.1%, maximum 37.1%.

### 3.3. Analysis of Squalene Content in Used Wipes

The 25 wipes used in the face of the volunteers were analyzed for squalene content. Prior to conducting the study representative samples of the wipes were analyzed using the same method to exclude contamination of the wipes with squalene. The result was < 0.05 mg/wipe (LOD of the method). After use a mean of 1.7 mg/wipe was determined (n = 25, RSD 54.5%), values ranged from 0.2 to 3.7 mg/wipe.

According to literature16 squalene content in human sebum is 12%–20%. 0.2 to 3.7 mg squalene/wipe would therefore be equivalent to 2 to 31 mg sebum / wipe (12% in sebum) or 1 to 18 mg sebum/wipe (20% in sebum). A mean sebum production rate in adults is approximately 1 mg/10 cm^2^ every 3 h [26].

### 3.4. Deposition of Niacinamide on Volar Forearms

Similar to face measurements at each point two measurements at the surface (0 µm) and at 5 µm were performed and the results were summarized. The mean of all measurement points was calculated and statistically evaluated.

For the placebo a mean result of 0 was calculated (N = 25). The active showed a mean result of 27.54 (N = 25, SD = 30.72), values ranged from 0 to 145.77. Three subjects showed a result of 0 (#12: female, 46 years, African, #17: female, 36 years, African, #22: male, 32 years, African).

## 4. Discussion

### 4.1. Deposition of Niacinamide on Facial Skin

The data demonstrate statistically significant (*p* < 0.05) the successful deposition of niacinamide.

Table 1 shows the results of the statistical analysis of the study population as well as per sex and gender. 

Except for one subject prior to application no niacinamide was detected, the Raman result was 0. Therefore, there is very little variation in the data set prior to application. The data set after application shows a considerable variation. Consequently, Levene’s test for equality of variances is statistically significant, which indicates that the group variances are unequal in the population and all sub groups evaluated. Therefore, evaluation is not performed using the pooled estimate for the error term for the t-statistic. Instead an adjustment to the degrees of freedom using the Welch-Satterthwaite method is performed. As a result of not being able to assume equal variances there is a reduction in the value of the t-statistic and a large reduction in the degrees of freedom (df). This has the effect of increasing the p-value. However, the difference between the Raman result prior and after application is statistically significant (*p* < 0.05) in all subjects as well as all sub groups analyzed (male, female, Caucasian, Asian, African). 

It is very evident that Raman measurement is more difficult in African skin. One African subject shows relatively high Raman data prior to application. Another African subject shows Raman results of 0 before and after application (subject #9, female, 22 years). However, analysis of the used wipe for residual niacinamide content reveals a decrease of 28.0%, almost equal to the mean decrease of 28.3%. Therefore, it can be concluded that niacinamide is deposited but Raman analysis could not detect it. Especially the evaluation of face profiles is found to be more difficult in African subjects (skin type V and VI according to Fitzpatrick classification). There is a substantial fluorescence of melanin observed that interfered with the signal of niacinamide. Depending on the wavelength of the laser used fluorescence of melanin is either visible as intense and broad peaks at about 1580 and 1380 cm^−1^ (excitation wave length of 457.9 nm and 514.5 nm, respectively) or as broad noise (excitation wave length of 785 nm) [27]. For the present study an instrument using a 785 nm laser was used. With increasing concentration of melanin in the skin the background noise caused by melanin fluorescence made the detection of niacinamide more difficult. The amount of melanin is correlated with the skin type. As a consequence, the Raman signal can be inconclusive in as far as sometimes a signal can be observed and sometimes not. Therefore, the problem is only observed in subjects with skin type Fitzpatrick V/VI.

However, the difference in the Raman signal prior and after application of the wipe is also significant for the African subjects without exclusion of any data.

### 4.2. Analysis of Residual Niacinamide Content in Used Wipes

Almost 30% of the niacinamide was liberated. Both gender showed considerable liberation (26.6% female, 29.9% male subjects). A statistically significant difference between the genders is confirmed.

Results of the statistical analysis of some sub groups are presented in Table 2. Statistical evaluation is performed using independent *t*-test. 

From the result of Levene’s Test for equality of variances, the null hypothesis that there is no difference in the variances between the groups is accepted and the alternative hypothesis that there is a statistically significant difference in the variances between groups is rejected (sig > 0.05 i.e., *p* < 0.05).

The *t*-test for differences of means is only found significantly different between male and female (sig < 0.05 i.e., *p* < 0.05). Sub group analysis of different ethnicities shows no statistically significant difference (sig > 0.05 i.e., *p* < 0.05). The amount of niacinamide liberated from the wipes for male subjects is significantly higher than for female subjects. Possibly male skin in the face is more uneven compared to female skin (e.g., more hair, more wrinkles) [28]. In the different ethnicities the differences are too small to be statistically significant.

### 4.3. Analysis of Squalene Content in Used Wipes

Results of statistical sub group analysis are presented in Table 3. 

The cleansing effect of the wipe is successfully shown by using uptake of squalene as surrogate parameter. Squalene is found in wipes used on male skin as well as female skin. On male skin more squalene is found on a statistically significant level (*p* < 0.05). 

Statistical evaluation is performed using independent *t*-test. From the result of Levene’s Test for equality of variances, the null hypothesis that there is no difference in the variances between the groups is accepted and the alternative hypothesis that there is a statistically significant difference in the variances between groups is rejected (sig > 0.05 i.e., *p* < 0.05).

The *t*-test for differences of means is found significantly different between male and female and between Caucasian and Asian subjects. (sig < 0.05 i.e., *p* < 0.05). The amount of squalene taken up from male subjects is much higher than from female subjects. This might be due to females cleansing their face more thoroughly compared to males. In the literature studies have demonstrated conflicting outcomes, both supporting and rejecting gender-specific variation [29].

A significant difference is detected between Caucasian and Asian subjects with almost double the amount of squalene removed from the Caucasian subjects. Sub group analysis between Caucasian and African subjects on one side and Asian and African subjects on the other side show no statistically significant difference (sig > 0.05 i.e., *p* < 0.05). Very limited information is available in the literature regarding the sebum production in different ethnicities. Some studies have shown a larger sebum secretion in African subjects compared to Caucasian subjects. Other studies have shown no differences between Caucasian, Asian and African subjects [25,30].

### 4.4. Deposition of Niacinamide on Volar Forearms

Table 4 shows the results of the statistical analysis of the study population as well as per sex and gender. Niacinamide result for placebo wipe was 0 in all subjects. Therefore, it can be concluded that treatment of the skin with the microfiber wipe does not result in erroneous detection of niacinamide.

Because of the zero variation in the placebo data Levene’s tests for equality of variances are statistically significant for the complete study population as well as for gender and ethnicity, which indicates that the group variances are unequal in the population and all sub groups evaluated. Therefore, *t*-test is performed using the algorithm for unequal variances.

The difference between the Raman result in placebo and active is statistically significant (*p* < 0.05) in all subjects as well as all sub groups analyzed except for African subjects. The main reason for failure in demonstrating a statistically significant difference between placebo and active in the African sub group are the three out of six subjects where no niacinamide is detected following use of active wipe. As a result the mean result is by far the lowest in all sub groups analyzed. Together with the low degree of freedom due to limited number of subjects in this group the calculated two tailed significance factor is greater than 0.05. As already discussed previously, this is mainly a problem of interference of melanin with the signal of niacinamide. The number of African subjects is already limited (N = 6), therefore no attempt has been made to exclude the three subjects showing no niacinamide signal after use of active wipe. In order to obtain statistically significant results in African skin more subjects would be required.

## 5. Conclusions

It could be demonstrated that a novel technology, based on a microfiber tissue loaded with liposomized actives (e.g., niacinamide), is able to successfully transfer substantial amounts of the active substance to the skin when moistened and to cleanse the skin at the same time.

The data clearly show that the delivery is not negatively impacted by the cleansing capacities of the wipe.

The results are independent of gender and ethnicity. 

Technology inherent no excipients or additives are required resulting in lower allergenic potential and more simple formulation development. Both aspects make it a very interesting alternative to conventional delivery technologies like creams or serums.

## Figures and Tables

**Table 1:** Statistical analysis of niacinamide content obtained from the face.

		Niacinamide [a.u.]			Levene’s Test			*t*-Test for Equality of Means		
Complete Study Population	N	Mean	SD	Std Error of the Mean	F	Sig	Var. Hom.	t	df	Sig (2 Tailed)
prior application	25	0.68	3.39	0.68	37.59	0.000	No	−8.23	24.63	0.000
post application	25	49.77	29.64	5.93						
Female subjects	N	Mean	SD	Std error of the mean	F	Sig	Var. Hom.	t	df	Sig (2 tailed)
prior application	12	0	0.00	0.00	15.10	0.001	No	−5.70	11.00	0.000
post application	12	50.94	30.97	8.94						
Male subjects	N	Mean	SD	Std error of the mean	F	Sig	Var. Hom.	t	df	Sig (2 tailed)
prior application	13	1.31	4.71	1.31	20.28	0.000	No	−5.70	12.61	0.000
post application	13	48.7	29.58	8.21						
Caucasian subjects	N	Mean	SD	Std error of the mean	F	Sig	Var. Hom.	t	df	Sig (2 tailed)
prior application	13	0	0.00	0.00	29.46	0.000	No	−6.57	12.00	0.000
post application	13	52.93	29.04	8.05						
Asian subjects	N	Mean	SD	Std error of the mean	F	Sig	Var. Hom.	t	df	Sig (2 tailed)
prior application	6	0	0.00	0.00	6.16	0.032	No	−6.22	5.00	0.002
post application	6	68.28	26.88	10.97						
African subjects	N	Mean	SD	Std error of the mean	F	Sig	Var. Hom.	t	df	Sig (2 tailed)
prior application	6	2.83	6.93	2.83	5.35	0.043	No	−3.00	6.77	0.021
post application	6	24.43	16.20	6.61						

**Table 2:** Statistical sub-group analysis of residual niacinamide in wipes.

		% Nicotinamide Released			Levene’s Test			*t*-Test for Equality of Means		
Subgroup	N	Mean	SD	Std Error of the Mean	F	Sig	Var. Hom.	t	df	Sig (2 Tailed)
Male	13	29.92	3.86	1.07	2.69	0.115	Yes	−2.55	23.00	0.018
Female	12	26.58	2.50	0.72						
Caucasian	13	28.58	3.26	0.90	0.60	0.451	Yes	1.39	17.00	0.181
Asian	6	26.45	2.66	1.08						
Caucasian	13	28.58	3.26	0.90	1.38	0.256	Yes	−0.56	17.00	0.583
African	6	29.63	4.94	2.02						
Asian	6	26.45	2.66	1.08	2.17	0.172	Yes	−1.39	10.00	0.195
African	6	29.63	4.94	2.02						

**Table 3:** Statistical sub-group analysis of squalene uptake by the wipes.

		Squalen [mg/wipe]			Levene’s Test			*t*-Test for Equality of Means		
Subgroup	N	Mean	SD	Std Error of the Mean	F	Sig	Var. Hom.	t	df	Sig (2 Tailed)
Male	13	2.05	0.79	0.22	0.17	0.687	Yes	−2.51	23.00	0.020
Female	12	1.23	0.84	0.24						
Caucasian	13	2.01	0.79	0.22	0.03	0.865	Yes	2.58	17.00	0.020
Asian	6	1.01	0.77	0.31						
Caucasian	13	2.01	0.79	0.22	0.83	0.375	Yes	1.19	17.00	0.250
African	6	1.51	0.97	0.39						
Asian	6	1.01	0.77	0.31	1.07	0.325	Yes	−1.00	10.00	0.343
African	6	1.51	0.97	0.39						

**Table 4:** Statistical analysis of niacinamide content obtained from volar forearms.

		Niacinamide [a.u.]			Levene’s Test			*t*-Test for Equality of Means		
Complete Study Population	N	Mean	SD	Std Error of the Mean	F	Sig	Var. Hom.	t	df	Sig (2 Tailed)
Placebo	25	0	0.00	0.00	22.42	0.000	No	−4.48	24.00	0.000
Active	25	27.54	30.72	6.14						
Female subjects	N	Mean	SD	Std error of the mean	F	Sig	Var. Hom.	t	df	Sig (2 tailed)
Placebo	12	0	0.00	0.00	29.48	0.001	No	−3.31	11.00	0.007
Active	12	20.4	21.36	6.17						
Male subjects	N	Mean	SD	Std error of the mean	F	Sig	Var. Hom.	t	df	Sig (2 tailed)
Placebo	13	0	0.00	0.00	7.96	0.009	No	−3.32	12.00	0.006
Active	13	34.12	37.04	10.27						
Caucasian subjects	N	Mean	SD	Std error of the mean	F	Sig	Var. Hom.	t	df	Sig (2 tailed)
Placebo	13	0	0.00	0.00	8.68	0.007	No	−3.80	12.00	0.003
Active	13	38.42	36.49	10.12						
Asian subjects	N	Mean	SD	Std error of the mean	F	Sig	Var. Hom.	t	df	Sig (2 tailed)
Placebo	6	0	0.00	0.00	16.19	0.002	No	−3.18	5.00	0.024
Active	6	26.04	20.03	8.18						
African subjects	N	Mean	SD	Std error of the mean	F	Sig	Var. Hom.	t	df	Sig (2 tailed)
Placebo	6	0	0.00	0.00	59.88	0.000	No	−2.15	5.00	0.084
Active	6	5.45	6.21	2.54						
African subjects	N	Mean	SD	Std error of the mean	F	Sig	Var. Hom.	t	df	Sig (2 tailed)
Placebo	5	0	0.00	0.00	26.60	0.001	No	−2.33	4.00	0.080
Active	5	6.54	6.27	2.80						
African subjects	N	Mean	SD	Std error of the mean	F	Sig	Var. Hom.	t	df	Sig (2 tailed)
Placebo	3	0	0.00	0.00	15.80	0.016	No	−6.92	2.00	0.020
Active	3	10.89	2.73	1.57						
